# Supplementary material for: Random access parallel microscopy
Source: eLife. 2021 Jan 12;10:e56426. doi: 10.7554/eLife.56426 (PMC7843131; doi:10.7554/eLife.56426)
Supplement: Supplementary file 1. [file elife-56426-supp1.zip › RAP_assembly/RAP stl files and assembly guide.pdf]

RAP stl files and assembly guide

The two configurations (Configuration 1 and Configuration 2) can be assembled using optomechanical components and 3D printed parts. Please see the main text for information regarding the cameras, lenses, and parabolic reflector.

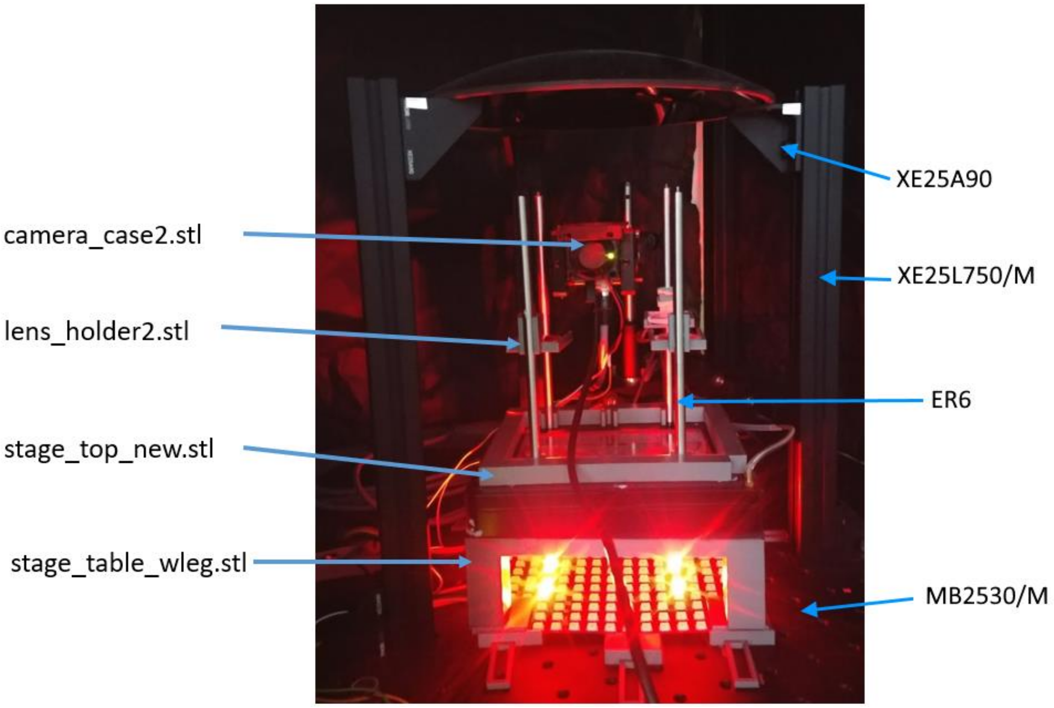

Configuration 1: This configuration uses 3D printed components that allow rough focus adjustment of up to six lenses (four shown). Lenses are held in a 3D printed holder (lens_holder2.stl) which attach to and can be positioned anywhere along a Thorlabs 6mm diameter metal rod (Thorlabs ER6). The parabolic reflector is suspended by three optical rails and brackets (Thorlabs XE25L750/M, XE25A90). The camera is removed from its original case and mounted in a 3D printed holder (camera_case2.stl) and positioned using 12.7mm optical posts (Thorlabs TR75/M, RA90, UPH4). The unit is mounted on a standard breadboard (Thorlabs MB2530/M). A stage-top incubator is used for samples that require temperature and humidity regulation. The camera is cooled by an external fan (Adafruit # 4468).

‘Configuration 2’ uses two 40 element LED arrays (Adafruit NeoPixel Shield for Arduino - 40 RGB LED Pixel Matrix, part #1430), which have a LED spacing compatible with 96 well plates. The LEDs come mounted on PCB boards that are too large to be tiled, so any configuration using these arrays cannot illuminate all 96 wells without moving the arrays or the 96 well plate. The camera is positioned between the two LED arrays to minimize the distance between the objective lenses and the sensor: as the camera sensor is mounted on a 29 mm square board, 4 additional wells are obscured by the camera for a maximum of 76 imaged wells using this configuration. The assembled unit is shown in Figure 1- figure supplement 1.

Wiring and programming instructions for the LED arrays can be found on the manufacturer’s website (https://learn.adafruit.com/adafruit-neopixel-uberguide). Positioning of the camera is achieved using two construction laser levels (Bosch GPL2): the camera is centered when the beams from the two vertically

aligned converge on the center of the sensor. The camera is attached to the computer with a USB3 cable and the Arduino Uno card via a 2-wire trigger cable: care must be taken that these cables do not inadvertently obscure wells.

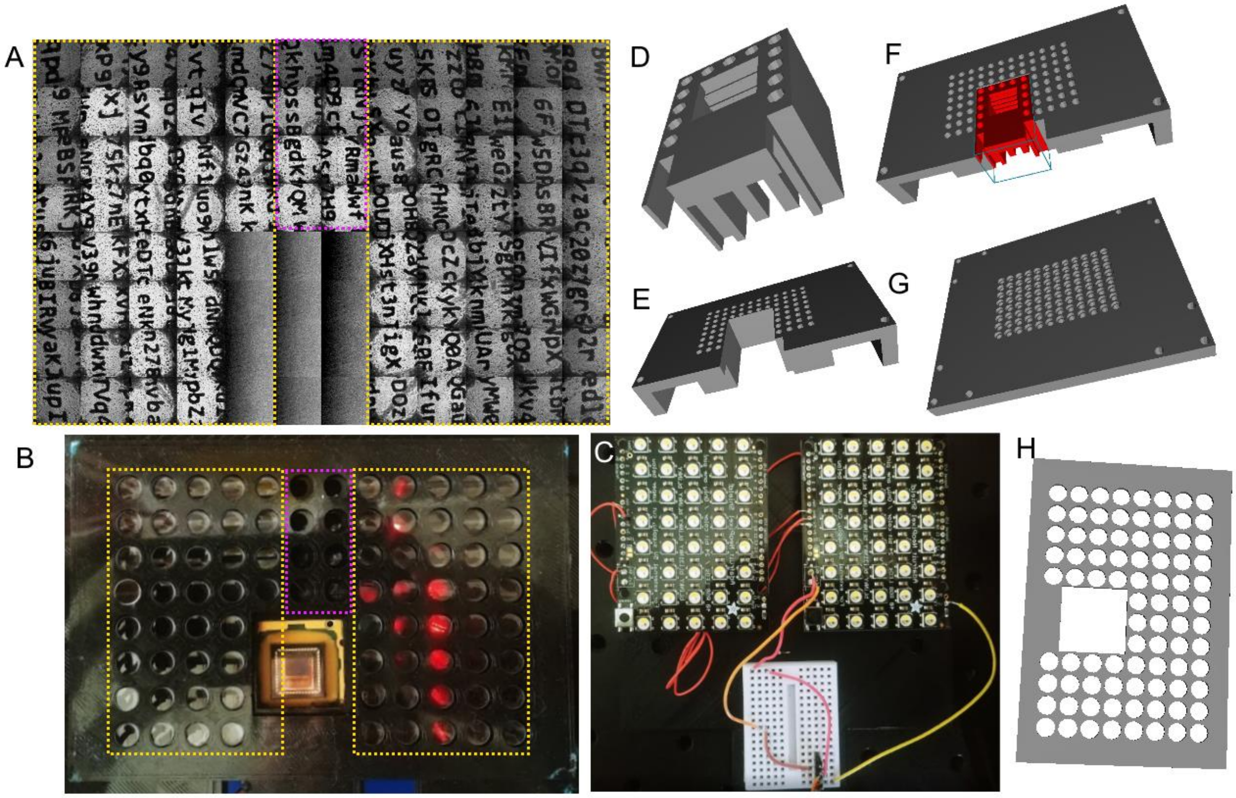

<u>Configuration 2:</u> (A) Calibration image of random letters (1.1mm high) printed on an acetate sheet: this image is a composite captured one row at a time, with the LED array being repositioned relative to the objective lenses to capture images from 84 wells. The wells outlined in magenta are not addressable without moving the LED array. The twelve wells obscured by the camera are shown as grey squares. The position of the two LED arrays relative to the sensor are outlined in yellow. (B) The main 3D printed lens and camera holder, shown from the top, with colored outlines showing addressable (yellow) and non-addressable (magenta) regions (see panel ‘f’ for a view of the 3D printed component). (C) The two LED arrays along with a small solderless breadboard used to simplify connections. The units are controlled by an Arduino Uno microcontroller (not shown) (D) The 3D printed camera holder (‘camera_holder_v3.stl’) holds the camera board and 13 lenses, as well as a small fan (Adafruit part #4468). (E) The camera holder is positioned in the main lens holder and held in place by friction (‘96wellholder_base.stl’), as shown in (F). This component has four holes that allow it to be held in place by 6mm rods (Thorlabs ER6). The construction also includes 2 larger plates with 96 holes (‘ninetysix_well_main.stl’) which contain guide holes for 6mm rods. (H) The lenses are positioned using a thin guide (‘lens_top_guide.stl’). Components for holding the parabolic reflector are the same as those used in configuration 1.
